# A Novel Method to Establish a Rat ED Model Using Internal Iliac Artery Ligation Combined with Hyperlipidemia

**DOI:** 10.1371/journal.pone.0102583

**Published:** 2014-07-21

**Authors:** Chao Hu, Feixiang Wang, Yehao Dong, Jican Dai

**Affiliations:** 1 Departments of Urology, Affiliated Ren Ji Hospital, School of Medicine, Shanghai Jiao Tong University, Shanghai, P.R. China; 2 Institute of Forensic Science, National Ministry of Justice, Shanghai Key Laboratory of Forensic Medicine, Shanghai, P.R. China; Max-Delbrück Center for Molecular Medicine (MDC), Germany

## Abstract

**Objective:**

To investigate a novel method, namely using bilateral internal iliac artery ligation combined with a high-fat diet (BCH), for establishing a rat model of erectile dysfunction (ED) that, compared to classical approaches, more closely mimics the chronic pathophysiology of human ED after acute ischemic insult.

**Materials and Methods:**

Forty 4-month-old male Sprague Dawley rats were randomly placed into five groups (n = 8 per group): normal control (NC), bilateral internal iliac artery ligation (BIIAL), high-fat diet (HFD), BCH, and mock surgery (MS). All rats were induced for 12 weeks. Copulatory behavior, intracavernosal pressure (ICP), ICP/mean arterial pressure, hematoxylin-eosin staining, Masson's trichrome staining, serum lipid levels, and endothelial and neuronal nitric oxide synthase immunohistochemical staining of the cavernous smooth muscle and endothelium were assessed. Data were analyzed by SAS 8.0 for Windows.

**Results:**

Serum total cholesterol and triglyceride levels were significantly higher in the HFD and BCH groups than the NC and MS groups. High density lipoprotein levels were significantly lower in the HFD and BCH groups than the NC and MS groups. The ICP values and mount and intromission numbers were significantly lower in the BIIAL, HFD, and BCH groups than in the NC and MS groups. ICP was significantly lower in the BCH group than in the BIIAL and HFD groups. Cavernous smooth muscle and endothelial damage increased in the HFD and BCH groups. Cavernous smooth muscle to collagen ratio, nNOS and eNOS staining decreased significantly in the BIIAL, HFD, and BCH groups compared to the NC and MS groups.

**Conclusions:**

The novel BCH model mimics the chronic pathophysiology of ED in humans and avoids the drawbacks of traditional ED models.

## Introduction

Erectile dysfunction (ED) is the consistent or recurrent inability to achieve and/or maintain a penile erection sufficient for satisfactory sexual performance [1]. Recent epidemiological reports showed that depending on the definition used and study design, ED prevalence ranged from 10% to 52%, and the annual incidence is 30 new cases per 1000 inhabitants in western countries [2]. Even though numerous ED animal models have been established, such as those involving hyperlipidemia, internal iliac artery ligation, diabetes mellitus, denervation, hypertension, smoking, pelvic irradiation and prostanoids, the arteriogenic ED model remains the most common type of model [3]–[10]. One reason for the popularity of the arteriogenic ED model is that performing internal iliac artery ligation is quite simple. However, the disadvantages of establishing arteriogenic ED models are the long duration of high-fat diet feeding and the auto-compensation observed 3 or more months after injury [11]–[13]. Another disadvantage is the high price of the special rats required for arteriogenic ED models, such as testosterone-supplemented spontaneously hypertensive rats, transgenic rats even though the period in ED developing was shorter, and rats with injuries made in the acute phase [14]–[16]. These disadvantages have slowed the study of the mechanisms of normal erection and the investigation of pathophysiological factors associated with ED.

Based on the classical methods used to establish ED animal models, this study aimed to avoid the long period required to establish ED with hyperlipidemia and auto-compensation after acute injury. The new ED model was established by combining bilateral internal iliac artery ligation and hyperlipidemia. To confirm that the new model was stable and mimicked the chronic pathophysiology of ED in humans, the model was assessed by the mating test, intracavernosal pressure examination, Masson's trichrome staining and endothelial nitric oxide synthase (eNOS) and neuronal nitric oxide synthase (nNOS) immunohistochemical staining.

## Materials and Methods

### Ethics Statement

The study was designed and conducted in accordance with the approval of the Animal Care and Use Committee of the School of Medicine, Shanghai Jiao Tong University.

### Animal Grouping and ED Model Establishment

Forty 4-month-old male Sprague Dawley rats were housed in a temperature controlled room with a 12:12-hour light: dark cycle and maintained with access to food and water *ad libitum*. After allowing the animals to become accustomed to the laboratory for 2 weeks, the rats were randomly divided into five groups. (A) The normal control group (NC, n = 8) received no surgery and was fed a normal diet containing 9.7% water, 20.5% protein, 4.62% lipid, 4.35% fibrin, 1.23% calcium, 0.91% phosphorus, 52.5% nitrogen free extract and 6.19% others. (B) The bilateral internal iliac artery ligation group (BIIAL, n = 8) received intraperitoneal anesthesia with sodium pentobarbital (30 mg/kg, ip) under aseptic conditions. A 6-0 wire was used to ligate the bilateral internal iliac arteries, and the rats were fed normally. (C) The high-fat diet(for hyperlipidemia) group (HFD, n = 8) was fed a high-fat diet containing 9.7% water, 21.29% protein, 19.73% lipid, 4.20% fibrin, 1.25% calcium, 1.01% phosphorus, 36.67% nitrogen free extract and 6.15% others (the mixture of 10% egg yolk, 8% lard, 0.2% propylthiouracil, 0.5% bile salt, 4.8% salt and normal diet), and the rats did not undergo surgery. (D) The BIIAL combined with HFD group (BCH, n = 8) was treated with bilateral internal iliac artery ligation and was fed the high-fat diet described above. (E) The mock surgery group (MS, n = 8) received intraperitoneal anesthesia with sodium pentobarbital like the BIIAL group. A lower abdominal incision was made in the MS group without any further surgery, and the rats were fed normally. The experimental protocal were all started at the end of adaptation and all rats were raised in a specific pathogen free (SPF) environment for 12 weeks.

### Assessment of ED

After 12 weeks of ED induction, the following tests were conducted: mating test and assessment of mean arterial pressure (MAP) and intracavernosal pressure (ICP).

#### Mating test

The sexual behavior of male rats was monitored by trained observers that were without knowledge of the experimental design. The mating test was conducted in a sound-attenuated, air conditioned room lit with a dim red light, during the early portion of the dark cycle. Single male rats were placed in rectangular glass observation cages (40 cm×50 cm×40 cm) and allowed to become accustomed to the test chamber for 5 min. Then a sexually receptive female rat was introduced into the cage, and the mating test started. The mount number (MN) and intromission number (IN) were measured as previously described by Zanoli [17]. Tests were normally ended immediately after reaching any of the following criteria: the first postejaculatory intromission, when intromission did not occur within 15 min, when ejaculation latency exceeded 30 min, or when the postejaculatory interval exceeded 15 min.

The rats in NC and MS groups which performed normal erectile function were picked out and those in BIIAL, HFD and BCH groups which performed weak erectile function were picked out to finish the following tests.

#### Assessment of MAP and ICP

Rats were anesthetized with sodium pentobarbital (30 mg/kg, ip), and anesthesia was maintained with supplemental sodium pentobarbital as needed. The shaft of the penis was freed of skin and fascia, and the right corpus cavernosum was cannulated by insertion of a 30-gauge needle connected to a pressure transducer to assess ICP, and the left carotid artery was cannulated with a PE-50 catheter filled with 250 IU/ml heparinized saline to measure MAP. Continuous monitoring of MAP and ICP was performed after the vasoactive drug apomorphine (APO, 100 µg/kg) was subcutaneously injected according to Heaton's method [18]. The data were collected for analysis using Polyview data-acquisition software (AstroMed).

### Identification of Hyperlipidemia

Twelve weeks after induction of hyperlipidemia, serum lipids were assayed. The blood of rats were obtained by cardiac puncture and were collected into centrifuge tubes, and serum was prepared by centrifugation (3000 rpm, 20 min at 4°C) and stored frozen until the assessment. Total cholesterol (TC), triglycerides (TG), and high-density lipoproteins (HDL) were assayed on an automated analyzer (Roche Cobas-Mira) utilizing commercially available kits according to the manufacturer's instructions and run procedures [19].

### Histological Analysis

The rat corpus cavernosum was fixed in cold fresh 2% formaldehyde in 0.1 M phosphate buffer (pH 7.4) for 4 hours, cryoprotected in 15% sucrose for 20 hours at 4°C, and then embedded in paraffin. The paraffin-embedded tissues were cut into 5 µm sections and adhered to superfrost plus slides. Sections of rat corpus cavernosum were deparaffinized and rehydrated. The sections were then used for hematoxylin-eosin (HE) staining, Masson's trichrome staining, or endothelial nitric oxide synthase (eNOS) and neuronal nitric oxide synthase (nNOS) immunohistochemical staining.

#### Hematoxylin-eosin (HE) staining

After being labeled, the sections were stained with HE (artificial hematoxylin and eosin) method. Histopathological changes in the corpus cavernosum, cavernous smooth muscle, and endothelium thickness were observed.

#### Masson's trichrome staining

After being labeled, the sections were stained with Masson's trichrome method which was used to distinguish and analyze for cavernous smooth muscle (stained in red) and collagen (blue) and expressed as the ratio of cavernous smooth muscle/collagen. Slides were digitized using a Zeiss Axioplan 2 microscope connected to AxioCam digital camera with KS400 image analysis (Version 3.0) software. Quantitative image analysis was performed by KS400 image analysis software, which recognized the image and obtained the ratio by RGB program.

#### eNOS and nNOS immunohistochemical staining

After being labeled, sections were immersed in ethylenediaminetetraacetic acid (EDTA) (for nNOS) or citric acid (for eNOS) antigen retrieval solution. Endogenous peroxidases were quenched by incubation with 7.5% hydrogen peroxide in water for 5 min. The slides were incubated in a blocking solution (10% goat serum in 0.1 M phosphate buffered saline [PBS, pH 7.4] and 0.3% Triton X-100) for 20 min. For nNOS immunohistochemical staining, the sections were incubated with a rabbit polyclonal anti-nNOS IgG antibody (catalog#4236, Cell Signaling Technology) at a dilution of 1∶100 in blocking solution. For eNOS immunohistochemical staining, the sections were incubated with a rabbit polyclonal anti-eNOS IgG antibody (catalog# sc-654, Santa Cruz Biotechnology, Santa Cruz) at a dilution of 1∶100 in blocking solution. For both eNOS and nNOS immunohistochemical staining, biotinylated goat anti-rabbit IgG (1∶200, Vector Labs) was used as the secondary antibody. Binding of the secondary antibody was visualized with streptavidin horseradish peroxidase (DAKO) and diaminobenzidine (Vector Labs), which yields a brown product. Between all reaction steps, the slides were rinsed with 0.1 M PBS (pH 7.4). Counterstaining was performed using Mayer's hematoxylin. Slides were digitized and analyzed by KS400 image analysis software. The KS400 image analysis software transformed the image to a grey scale interval from 0 to 250 units, which was expressed as a positive rate (occupation of total area and total intensity of NOS in the whole image, grey scale: 10 to 110 units) or a strong positive rate (grey scale: 10 to 80 units) [20].

### Statistical Analysis

All data were presented as the mean ± standard deviation (SD). One-way analysis of variance was used to compare the means of data among groups. Bonferroni's test was applied to assess differences between two groups. A *p*-value <0.05 was considered significant. The analysis was performed by SAS 8.0 for Windows.

## Results

### Body Weight of 5 Groups

At the start of experiment, body weight of each group did not differ significantly with each other. During the period, body weight of all groups elevated. At the end of experiment, body weight of HFD and BCH groups significantly higher than that of other groups as shown in Table 1.

**Table 1:** Changes of body weight of 5 groups.

Group	Body Weight(g, 4 mons)	Body Weight(g, 7 mons)
NC	497±35	668±20
BIIAL	489±54	653±47
HFD	483±32	743±37 *
BCH	495±28	705±39 *
MS	490±43	650±40

* *p*<0.05 vs. other groups.

### Assessment of ED

In the mating test, mounting was observed in all rats in the NC group and 7 rats in the MS group, and intromission was observed for 5 rats in both the NC and MS groups; however, no rat in the BCH group showed mounting or intromission. In the BIIAL group, mounting was observed for 3 rats, and no intromission was observed. In the HFD group, mounting was observed for 6 rats, and intromission was observed for 3 rats. Thus, 5 rats in NC, 8 rats in BIIAL, 5 rats in HFD, 8 rats in BCH and 5 rats in MS were picked out to finish the following tests.

**Figure 1:**
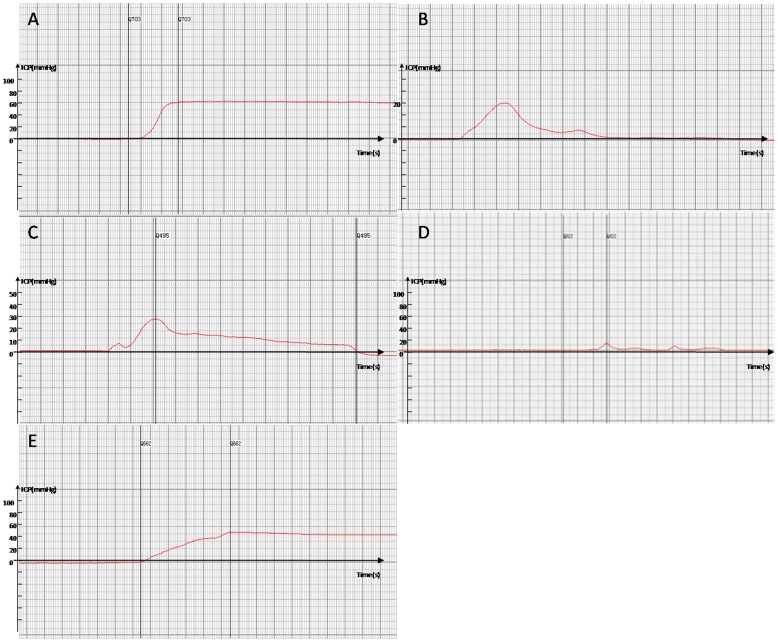
The ICP levels of NC, BIIAL, HFD, BCH and MS groups after 12 weeks. (A) In the NC group, the ICP in the rat corpus cavernosum increased greatly during a normal erection induced by apomorphine and lasted for a relatively long period. (B) In the BIIAL group, the ICP in the rat corpus cavernosum showed a low increase and short duration during an invalid erection. (C) In the HFD group, the ICP in the rat corpus cavernosum showed a low peak but a relatively long duration during an invalid erection. (D) In the BCH group, the ICP in the rat corpus cavernosum showed an extremely slow increase and minor peak, and almost no duration for the erection was identified. (E) In the MS group, the ICP in the rat corpus cavernosum showed a rapid increase and a long duration similar to the NC group.

In the ICP and ICP/MAP assessment, total ICP and ICP/MAP were decreased in the BIIAL, HFD, and BCH groups when compared to the NC and MS groups as shown in Table 2. The erection duration was significantly shorter in the experimental groups (BIIAL, HFD, and BCH) compared to the NC group. The difference between the NC and MS groups were not significant. The ICP and erection duration level of the BCH group was lower than that of the BIIAL and HFD groups (*p*<0.05) as shown in Table 2 and Figure 1.

**Table 2:** Mount number, intromission number, increase in ICP and ICP/MAP and duration.

Group	MN	IN	Increase in ICP (mmHg)	MAP (mmHg)	ICP/MAP	Duration(s)
NC	8	5	65.78±12.84 #	82.22±6.48	0.79±0.16 #	65.6±5.9 #
BIIAL	3	0	19.24±7.74 * #	83.60±7.46	0.23±0.09 *	36.3±4.3 * #
HFD	6	3	24.84±10.22 * #	87.80±5.88	0.30±0.12 * #	48.8±7.5 * #
BCH	0	0	6.42±2.40 *	85.24±6.52	0.07±0.03 *	28.7±3.8 *
MS	7	5	58.96±9.66 #	83.04±6.46	0.71±0.12 #	70.4±6.8 #

* *p*<0.05 vs. NC group;

# *p*<0.05 vs. BCH group.

### Identification of Hyperlipidemia

Twelve weeks after the induction of hyperlipidemia, the concentrations of TC and TG in the HFD and BCH groups were significantly higher than in the NC, BIIAL, and MS groups as shown in Table 3. Though the serum TC and TG concentrations in the BCH group were significantly lower when compared to the HFD group, only the difference in TC was significant. The serum HDL concentration was significantly lower in the HFD group and BCH group than in the NC, BIIAL, and MS groups. Furthermore, the level of HDL in the BCH group was lower than that of the HFD group (*p*<0.05). The serum lipids did not differ significantly between the NC, BIIAL, and MS groups. These results are shown in Table 3.

**Table 3:** Serum lipid concentrations.

Group	TC (mmol/L)	TG (mmol/L)	HDL (mmol/L)
NC	1.82±0.24 #	0.75±0.16 #	2.65±0.37 #
BIIAL	1.78±0.33 #	0.71±0.10 #	2.51±0.43 #
HFD	5.27±0.25 *	1.27±0.21 *	1.65±0.25 *
BCH	3.15±0.18 * #	1.12±0.23 *	1.15±0.14 * #
MS	1.79±0.21 #	0.73±0.17 #	2.50±0.47 #

* *p*<0.05 vs. NC group and BIIAL group;

# *p*<0.05 vs. HFD group.

TC  =  total cholesterol; TG  =  triglyceride; HDL  =  high density lipoprotein.

### Histological Analysis

**Figure 2:**
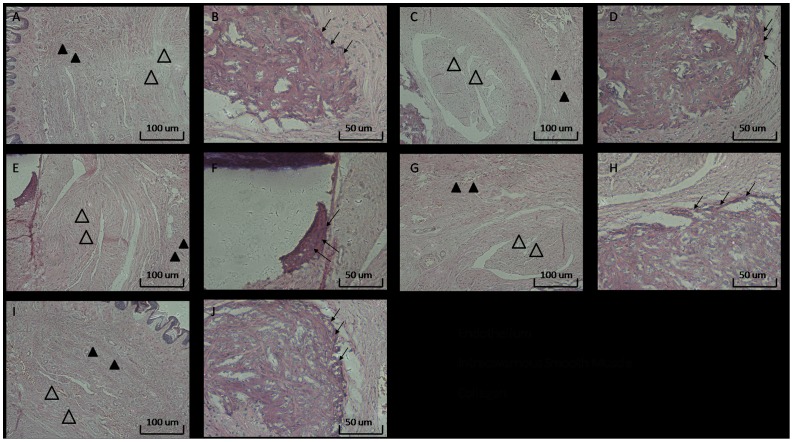
The histological analysis of rat corpus cavernosum in NC, BIIAL, HFD, BCH and MS groups after 12 weeks. Sections were made transversal to the penile axis. HE staining showed the changes in the cavernous smooth muscle, collagen and endothelium in rat corpus cavernosum in the different groups. (A) and (B) show the appropriate appearance of the cavernous smooth muscle and intact endothelium of the rat cavernosum in the NC group. (C) and (D) show decreased cavernous smooth muscle content and moderately damaged endothelium in the BIIAL group. (E) and (F) show increased cavernous smooth muscle content and a thickened endothelium in the HFD group. (G) and (H) show decreased cavernous smooth muscle content and severely damaged endothelium in the BCH group. (I) and (J) show the similar normal appearance of the cavernous smooth muscle and intact endothelium of the rat cavernosum in the MS and NC groups. A, C, E, G, original magnification ×100. B, D, F, H, original magnification ×200.

HE staining showed the pathological changes in the rat cavernosum, cavernous smooth muscle, and endothelium as shown in Figure 2. In the NC group, the cavernous smooth muscle had a moderate alignment, and the endothelium was intact (Figure 2A and 2B). Figure 2C shows the cavernous smooth muscle from the BIIAL group. The cavernous smooth muscle content in the BIIAL group decreased when compared to the NC group (data not shown, original magnification ×100). The integrity of the endothelium was damaged in the BIIAL group, as shown in Figure 2D. In the HFD group, the pathological changes are shown in Figure 2E. The cavernous smooth muscle content in the HFD group increased and the endothelium was thicker than in the NC group shown in Figure 2F. Figures 2G and 2H show the histological changes in the BCH group. The cavernous smooth muscle in the BCH group decreased, and the endothelium was severely damaged. There was no significant change in the MS group, which performed as well as the NC group. (Figure 2I and 2J)

**Figure 3:**
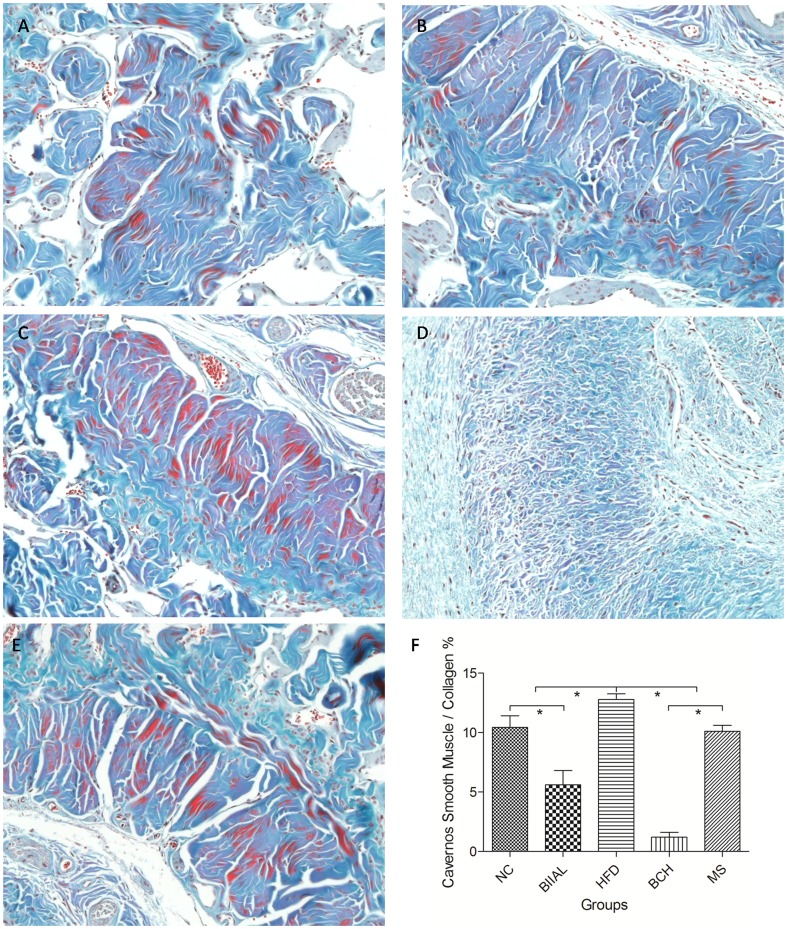
The Masson's trichrome staining of rat corpus cavernosum in NC, BIIAL, HFD, BCH and MS groups after 12 weeks. Sections were made transversal to the penile axis. Masson's trichrome staining showed the changes in the cavernous smooth muscle and collagen in rat corpus cavernosum in the different groups. (A) Normal cavernous smooth muscle and collagen expression was observed in the NC group. (B) Moderately decreased cavernous smooth muscle and increased collagen expression was observed in the BIIAL group. (C) Significantly increased cavernous smooth muscle expression was observed in the HFD group. (D) Significantly increased collagen and decreased cavernous smooth muscle expression was observed in the BCH group. (E) Similar cavernous smooth muscle and collagen expression was observed in the MS and NC groups. (F) shows the quantitative analysis results of cavernous smooth muscle to collagen ratio in different groups (* *p*<0.05).

Furthermore, with Masson's trichrome staining, Figure 3 shows the change of cavernous smooth muscle and collagen content in different groups. Using quantitative analysis, the cavernous smooth muscle to collagen ratio in the BIIAL (5.62±1.19%) and BCH (1.22±0.40%) groups were significantly lower than that in the HFD (12.79±0.47%), NC (10.44±0.98%) and MS (10.12±0.49%) groups. And the ratio in HFD group was significantly higher than that in NC and MS groups and the ratio in BCH group was significantly lower than that in BIIAL group as shown in Figure 3.

Table 4 shows the nNOS and eNOS immunohistochemical staining results. The BCH group had the lowest positive rate of both nNOS and eNOS staining. The positive rates of nNOS immunohistochemical staining in the BIIAL, HFD, and BCH groups were significantly lower than in the NC and MS groups. When compared to the BCH group, the positive rate of nNOS immunohistochemical staining in the NC, HFD, and MS groups were significantly higher, but the positive rate of nNOS immunohistochemical staining in the BIIAL group was not significantly different. Furthermore, in the analysis of the strong positive rate of nNOS immunohistochemical staining, the BIIAL, HFD and BCH groups had significantly lower staining than the NC and MS groups. When compared to BCH group, the strong positive rates of nNOS immunohistochemical staining in the NC, HFD and MS groups were significantly higher, but the strong positive rate of nNOS immunohistochemical staining in the BIIAL group was not significantly different. The positive rates of eNOS immunohistochemical staining in the HFD and BCH groups were significantly lower than in the NC and MS groups. When compared to the BCH group, the positive rate of eNOS immunohistochemical staining in the NC, BIIAL, and MS groups were significantly higher, but the positive rate of eNOS immunohistochemical staining in the HFD group was not significantly different. Furthermore, in the analysis of the strong positive rate of eNOS immunohistochemical staining, the HFD and BCH groups had significantly lower staining than the NC and MS groups. When compared to BCH group, the strong positive rates of eNOS immunohistochemical staining in the NC, BIIAL and MS groups were significantly higher, but the strong positive rate of nNOS immunohistochemical staining in the HFD group was not significantly different. Comparison between the NC and MS groups did not show any significant difference in terms of nNOS and eNOS expression.

**Table 4:** nNOS and eNOS positive rates.

Group	nNOS		eNOS	
	Positive rate%	Strong positive rate%	Positive rate%	Strong positive rate%
NC	20.02±3.05 #	9.50±2.50 #	8.49±1.46 #	2.81±0.88 #
BIIAL	8.22±1.82 *	3.78±1.00 *	7.80±0.54 #	2.43±0.11 #
HFD	12.45±2.00 * #	6.20±1.03 * #	3.44±0.83 *	0.91±0.36 *
BCH	6.31±2.47 *	2.64±1.12 *	3.15±0.95 *	0.72±0.29 *
MS	19.92±3.87 #	9.41±2.45 #	8.43±1.71 #	2.77±0.34 #

* *p*<0.05 vs. NC group;

# *p*<0.05 vs. BCH group.

**Figure 4:**
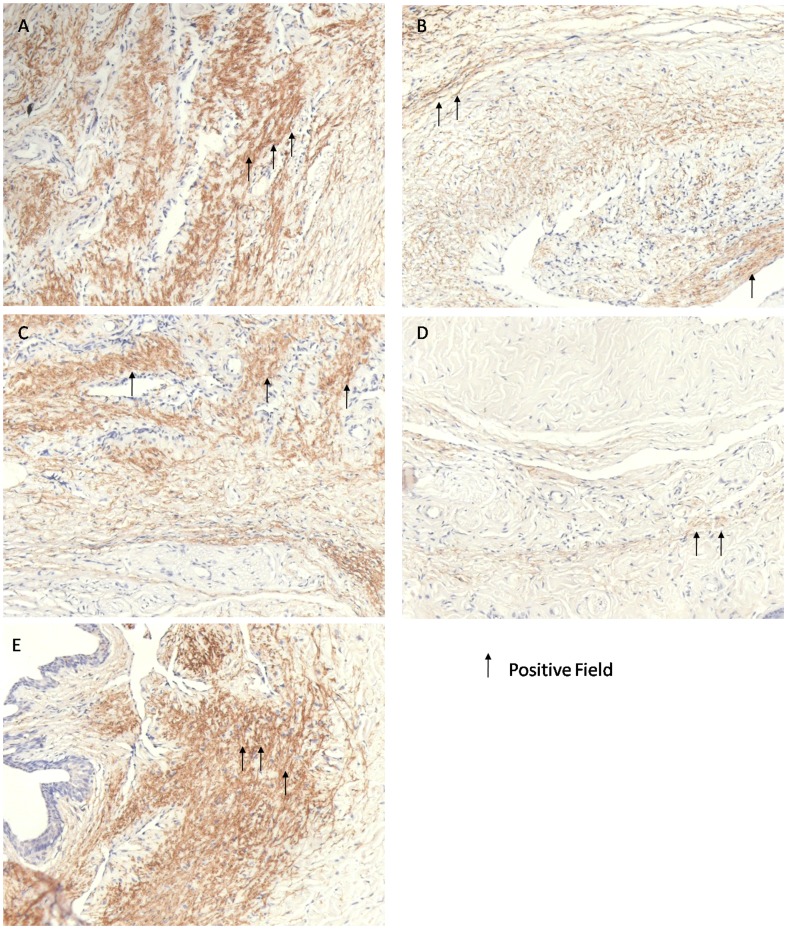
The nNOS immunohistochemical staining of rat corpus cavernosum in NC, BIIAL, HFD, BCH and MS groups after 12 weeks. Sections were made transversal to the penile axis. (A) Normal nNOS expression was observed in the NC group. (B) Moderately decreased nNOS expression was observed in the BIIAL group. (C) Mildly decreased nNOS expression was observed in the HFD group. (D) Significantly decreased nNOS expression was observed in the BCH group. (E) Similar nNOS expression was observed in the MS and NC groups.

**Figure 5:**
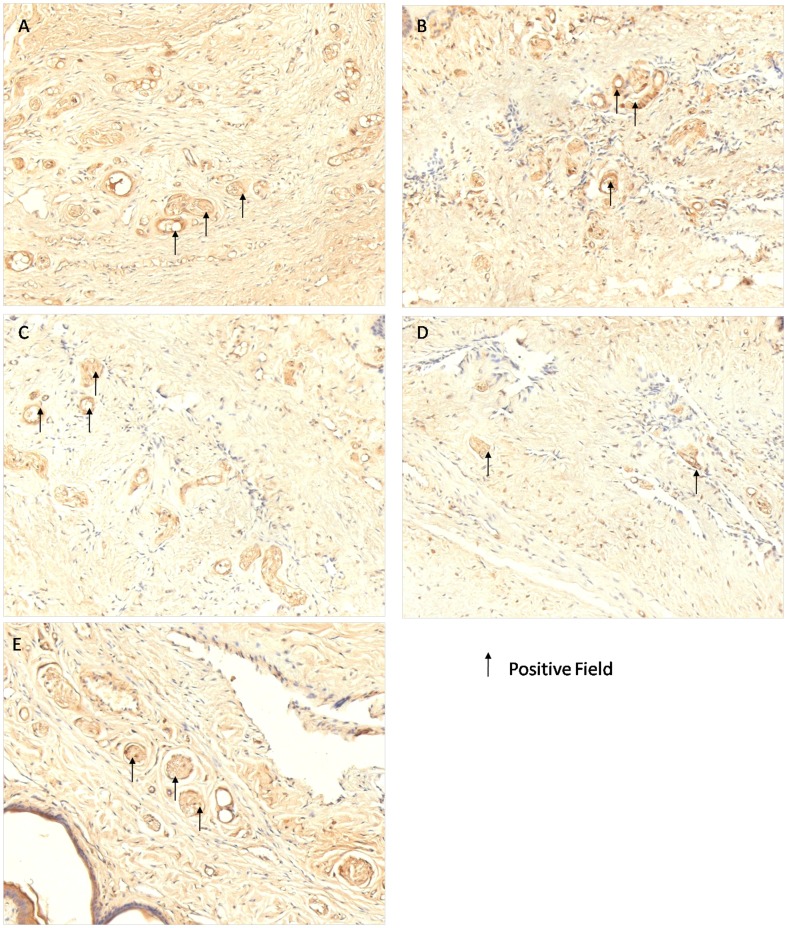
The eNOS immunohistochemical staining of rat corpus cavernosum in NC, BIIAL, HFD, BCH and MS groups after 12 weeks. Sections were made transversal to the penile axis. (A) Normal eNOS expression was observed in the NC group. (B) Mildly decreased eNOS expression was observed in the BIIAL group. (C) Severely decreased eNOS expression was observed in the HFD group. (D) Significantly decreased eNOS expression was observed in the BCH group. (E) Similar eNOS expression was observed in the MS and NC groups.

Importantly, the (strong) positive rate of nNOS immunohistochemical staining in the BIIAL group and the (strong) positive rate of eNOS immunohistochemical staining in the HFD group did not differ from the BCH group as shown in Table 4 and Figures 4 and 5.

## Discussion

The reduction in copulatory behavior, decrease in ICP and cavernous smooth muscle to collagen ratio and weakened expression of nNOS and eNOS support that a novel rat ED model was established by a method that combines bilateral internal iliac artery ligation with hyperlipidemia. Moreover, the lasting low expression of nNOS and eNOS in the rat ED model provides evidence that this model is durable.

In studying the mechanisms of ED, numerous animal models of ED have been established successfully, including hyperlipidemia, trauma, and arteriogenic models. Clinical epidemiological studies have shown that hyperlipidemia and pelvic fracture are risk factors of ED, and these factors interact with each other closely. However, previous studies concentrated on the risk factors independently without carrying out a comprehensive evaluation. For example, Bakircioglu reported that 4 months were necessary to establish a hyperlipidemia-induced ED model in rats, and Metze had reported severe sexual dysfunction after major pelvic trauma [11], [21]. Lee successfully developed a traumatic arteriogenic ED rat model, but ED may occur only in the acute phase and erectile function may show autocompensation at 6 weeks after surgery [12], [16]. Although no direct evidence, such as collateral circulation building or regrowth of new blood vessels, has demonstrated autocompensation occurred in the BIIAL, HFD and BCH groups at 12 weeks, the erectile function of these three groups decreased immediately after injuring according to previous report [12]. Indeed, our results indicate erectile function, such as ICP/MAP, nNOS and eNOS, in BIIAL and HFD groups are much better than that in BCH group, which hints that more erectile function is restored in BIIAL and HFD groups rather than that in BCH group, though the extent is modest, as well as Lee's work [12]. Future work concentrating on autocompensation at different time points would be expected to clarify the specific mechanism.

Previous reports showed that there was significantly more cavernous smooth muscle in hyperlipidemic rats than in normal rats [3], [20]. Moreover, hyperlipidemia may damage the endothelial barrier and expose the endothelium, especially the vascular smooth muscle, directly to the blood circulation [22], [23]. This exposure may give rise to the proliferation of vascular smooth muscle and result in the incrassation of endothelium with low endothelial cell content, suggesting that hyperlipidemia-induced ED may be the result of vascular blocking and cavernosal ischemia [23]. Our results show similar pathological changes in HFD group, and we find that cavernous smooth muscle to collagen ratio significantly decreased in BIIAL and BCH groups, seems paradoxical to the others. However, Yeşilli and Iacono et al. demonstrated the content of collagen increased and progressive fibrosis in hyperlipidemia induced ED rabbits and human cavernous tissue after one year observation and follow-up, suggesting that increase in collagen participates the pathology of ED when the modeling period extended [24], [25]. It is appropriate to infer that decrease in cavernous smooth muscle to collagen ratio is mainly depended on the collagen content, which relatively decreases the cavernous smooth muscle expression, in BCH group and the effect is strengthened by BIIAL injury.

Azadzoi et al. demonstrated reduced NOS activity may result in the impairment of endothelium-dependent, neurogenic relaxation in cavernosal tissue by balloon de-endothelialization and a high cholesterol diet [26]. In the present study, the expression of nNOS in the BIIAL and BCH groups was significantly decreased compared to the NC group, but the difference was not significant between the BIIAL and BCH groups. Furthermore, the expression of eNOS in the HFD and BCH groups was significantly decreased compared to the NC group, but there was no significant difference found between the HFD and BCH groups. According to a previous study, BCH may not reduce NOS subtype activity like HFD or BIIAL, but instead decrease the activity of NOS collectively [26]. As rats rarely develop atherosclerotic lesions like humans or rabbits, the method successful produced an ED rat model and reduced the model establishment period from 16 weeks to 12 weeks [27]. This method may be a feasible way to establish an ED model.

In addition, nNOS and eNOS initiate penile erection and are involved in sustaining the erection [28], [29]. Recently, many studies have associated eNOS and nNOS with the level of erectile function [4], [30]. Accordingly, BCH-induced ED showed lower ICP without recovery, and the NOS levels were consistently lower than normal. Thus, the BCH group may overcome the autocompensation of the BIIAL model and shorten the duration required to establish the HFD model, which further confirms our conclusion that the BCH model mimics the pathophysiology of ED in humans and avoids the drawbacks of traditional ED models.

Previous research showed that the serum lipid levels of animals fed a high-fat diet increased with the duration of feeding time and reached a plateau at 4 weeks, but vascular injury lagged. Hyperlipidemia-induced ED may occur and become stable at 4 months [11]. Clinically, chronic vascular injury is much more common than acute injury, and the chronic pathophysiology of ED should be emphasized. Therefore, we induced and maintained a chronic ED model for 12 weeks to establish the effect of hyperlipidemia fully.

Erectile function tests and histological evidence were used to verify that the novel BCH model reflected the pathophysiology of ED [20], [31]. This new method to establish a rat ED model shows several advantages over traditional methods. The new model not only mimics the pathophysiology of internal iliac artery ligation and hyperlipidemia, but it avoid the drawbacks and weaknesses of these methods, especially the long period required for establishing ED models involving acute injury.

Alvarenga et al. reported that training for sexual experience was needed to select rats that displayed favorable behavior [32]. However, as our study aimed to observe the existence of copulatory behavior rather than the level of sexual performance, we recorded the MN and IN to evaluate erectile function, as described by Zanoli et al. work, without training the rats [17]. This method provided clear results on whether the rats exhibited copulatory behavior.

The time point at which the ED model was stabilized was not evaluated in this study, and the model may stabilize at less than 12 weeks. Our future work will focus on determining the time point of stabilization to improve the methods for establishing rat ED models. Moreover, as hyperlipidemia has been widely and successfully applied in inducing ED in rabbits and mice with their features, our future research will apply this method in them and explore its features among them [24], [33], [34].

## Conclusions

This study was designed to seek a novel method to establish a rat ED model. By comparing the new BCH model with previous rat ED models, and evaluating these models with mating tests, ICP examination, Masson's trichrome staining and immunohistochemistry, the BCH model was shown to be an effective rat ED model. Moreover, the BCH model overcomes limitations of previous ED models. Future studies will determine the time points of the appearance and stabilization of ED in the BCH model.
